# Herbivore Immunity Exhibits a Biphasic Relationship with Plant Defense Toxin Concentrations

**DOI:** 10.1007/s10886-026-01772-w

**Published:** 2026-10-13

**Authors:** Enakshi Ghosh, Vincent S. Pan, Ian Pearse, Paul J. Ode

**Affiliations:** 1 Department of Entomology, Virginia Tech, Blacksburg, VA USA; 2 Department of Agricultural Biology, Colorado State University, Fort Collins, CO USA; 3 Department of Soil & Crop Sciences, Colorado State University, Fort Collins, CO USA; 4 Department of Integrative Biology, Michigan State University, East Lansing, MI USA; 5 W. K. Kellogg Biological Station, Michigan State University, Hickory Corners, MI USA; 6 Ecology, Evolution, and Behavior Program, Michigan State University, East Lansing, MI USA; 7 Fort Collins Science Center, U.S. Geological Survey, Fort Collins, CO USA; 8 Graduate Degree Program in Ecology, Colorado State University, Fort Collins, CO USA

**Keywords:** Tri-trophic interactions, Eco-immunology, Herbivory, Window of enhanced immunity, Plant defense, Hormesis

## Abstract

Plant chemical defenses are known to influence herbivore performance, yet their effects on herbivore immunity are poorly understood. In this study, we manipulated both the mean and variance of the concentration of a plant toxin in an artificial diet to assess the effects on herbivore immune function and survival. Our results revealed a biphasic immune response across increasing mean toxin concentrations, with exposure to low toxin concentrations enhancing immune function, creating a ‘window of enhanced immunity’, and exposure to higher concentrations suppressing it relative to toxin-free diets. Encountering variable toxin levels may alter this relationship by mixing toxin levels from within and outside this window. Importantly, we found that when the mean toxin concentration is just outside of the window of enhanced immunity, exposure to low variability in toxin concentrations enhanced herbivore immunocompetence compared to when they were exposed to high or no variation. Elevated immunity correlated with increased survival under pathogen challenge.

**Supplementary Information:**

The online version contains supplementary material available at https://doi.org/10.1007/s10886-026-01772-w.

## Introduction

Plant toxins have evolved, in part, as defenses against herbivores, yet herbivores often co-opt these toxins as protection against their own natural enemies, including pathogens, parasites, and predators. Positive effects of plant toxins on herbivore defenses are well-documented and include toxin sequestration for predator deterrence (Nishida 2002; Beran and Petschenka 2022) and self-medication behaviors to reduce pathogen and parasite loads (Singer et al. 2009, 2014). Recently, a handful of studies have documented the positive effects of plant toxins on herbivore immune systems (e.g., Garvey et al. 2021; Ghosh et al. 2023a, b) suggesting a novel mechanism by which herbivores can use plant toxins to protect themselves from their natural enemies.

Studies of the effects of plant toxins on herbivore immunity have shown conflicting patterns. On the one hand, several studies have shown that plant toxins reduce herbivore immunocompetence, a pattern that is often explained as a trade-off between the metabolic costs of detoxification and immune function (Smilanich and Muchoney 2022; Groen and Whiteman 2022). This idea, known as the “vulnerable host hypothesis” (Smilanich et al. 2009) or the “immunocompromised host hypothesis” (Reudler et al. 2011), assumes that plant toxins suppress herbivore immune responses. Yet, other studies have shown that plant toxins can enhance, rather than suppress, herbivore immunity against pathogens and parasitoids (e.g., Garvey et al. 2021; Ghosh et al. 2023a, b). Insect immunity operates through coordinated cellular and humoral defenses that detect, neutralize, and eliminate parasites, including parasitoids and pathogens. Cellular immunity involves hemocytes, including plasmatocytes, granulocytes, oenocytoids, and prohemocytes. Each hemocyte class has distinct functions: granulocytes play key roles in phagocytosis, engulfing and digesting pathogens; plasmatocytes are essential in initiating encapsulation (the formation of a cellular capsule around larger threats like parasitoid eggs); oenocytoids carry phenoloxidase (PO) enzymes for melanization; and prohemocytes have stem-cell like functions giving rise to other hemocyte classes. Complementing these cellular defenses are humoral responses that include the production of enzymes like PO, which participate in melanization of encapsulated objects (Hillyer 2016; Shikano 2017). Plant toxins may therefore alter herbivore immunocompetence by affecting one or more of these cellular and humoral components, with the direction of the effect potentially depending on toxin concentration.

The contrasting effects of plant toxins on herbivore immunity observed in previous studies raise the possibility that herbivores may enjoy enhanced immunocompetence when consuming lower concentrations of plant toxins but suffer suppressed immunocompetence when consuming higher concentrations. Such a biphasic relationship between plant toxins and herbivore immunity would be an example of hormesis, where low doses of an environmental stressor stimulate or enhance a biological response, and higher doses suppress or inhibit these functions (Mattson 2008; Calabrese 2005, 2009, 2014; Berry et al. 2020). Although the vast majority of studies have focused on human systems (e.g., Calabrese and Baldwin 2003; Slikker et al. 2004; Wan et al. 2024), a substantial number of studies have demonstrated the hormetic effects of environmental factors such as pesticides, radiation, and temperature on insect development and reproductive traits (Cutler et al. 2022). Yet, similar studies of the effects of plant toxins on insect immune systems are lacking. Recent studies have shown that different classes of plant toxins, including glucosinolates, alkaloids (esp., nicotine), and cardiac glycosides, can enhance the immune response of herbivorous insects (Garvey et al. 2021; Ghosh et al. 2022, 2023a, b a), but nothing is known about which doses represent a ‘window of enhanced immunity’ where toxins confer immune benefits at low concentrations (relative to a toxin-free diet), but suppress immune responses at higher concentrations for specific herbivores (Ode and Ghosh 2025).

Furthermore, an important complicating factor is the variability in the concentration of plant defenses encountered by the herbivore. Plant toxins are not evenly distributed within the plant. Their concentrations vary across plant tissues at very small spatial scales and fluctuate over time, exposing herbivores to varying toxin levels over the course of their development (Quintero et al. 2014; Ruhnke et al. 2009). Defense variability has been shown to suppress herbivore performance (Pearse et al. 2018; Holmes and Blubaugh 2023), alter herbivore feeding behavior (Pan et al. 2025a, b), and modulate herbivore-parasitoid interactions (Paul et al. 2021). Yet, despite the importance of understanding how herbivore immunity responds to plant toxins while simultaneously providing defense against their own natural enemies, the effects of plant toxin variability on herbivore immunity is unexplored. Two main outcomes may arise from exposure to variable toxin concentrations. First, variation in the toxin concentration an herbivore experiences may lead to a mismatch between toxin levels and herbivore physiological responses to those toxins (Pearse et al. 2018), including immunity and detoxification ability. In this scenario, herbivores might struggle to detoxify or tolerate varying toxin concentrations efficiently. This mismatch could suppress their immune responses and ultimately result in decreased fitness when attacked by parasites or pathogens. Second, herbivores may simply exhibit the average immunity across different levels of toxin (Wetzel et al. 2016). When an herbivore immune response to increasing toxin concentration is nonlinear, the average immunity across different levels of toxin is different from the immunity at the average level of toxin the herbivore experiences. This difference is known as the Jensen gap and serves as a null hypothesis for the effect of toxin variation (Pearse et al. 2018). Indeed, deviation from this null expectation has been used to detect the existence of nontrivial physiological processes, such as mismatched detoxification response (Pearse et al. 2018), that mediate the effect of toxin variation (Pan et al. 2025a, b).

In this study, we examined how changes in both the mean concentration and variation in a plant defense toxin influences herbivore immunity. We used artificial diets with varying concentrations of the furanocoumarin xanthotoxin, a plant toxin found in apiaceous host plants of the polyphagous caterpillar, the cabbage looper *Trichoplusia ni* (Lepidoptera: Noctuidae). By incorporating precise concentrations of xanthotoxin into the diet, we established controlled experimental conditions that alter the amount of toxin that herbivores are exposed to at levels that simulate the natural variability in toxin exposure. This system allowed us to explore three key questions: (1) how do increases in mean xanthotoxin exposure affect herbivore immunity, and is there a window of toxin concentrations that results in enhanced immune function?, (2) how does the herbivore immune system respond to fluctuations in exposure to xanthotoxin?, and (3) how do immunological changes induced by xanthotoxin mediate survival in the presence of pathogens?

## Methods

*Trichoplusia ni* is a polyphagous herbivore that has been recorded feeding on plants from over 40 families including the Apiaceae (Sutherland and Greene 1984). Some of these plants produce furanocoumarins such as xanthotoxin that function as defensive compounds against a broad array of herbivores. When feeding on these plants, caterpillars are naturally exposed to a wide range of furanocoumarin concentrations, including significant variation in xanthotoxin concentrations within individual plants, which can vary up to fourfold (Zangerl and Berenbaum 1994; Ode et al. 2004). Xanthotoxin is a well-studied phototoxic compound that binds DNA preventing transcription and translation, forming covalent cross-links and generating reactive oxygen species (Berenbaum 1981). Higher doses of xanthotoxin reduce *T. ni* performance, resulting in smaller body size, longer development times, and lower survivorship (Lampert et al. 2011; Pearse et al. 2018; Paul et al. 2021).

We maintained a laboratory colony of *T. ni* in an environmental chamber set at 25 ± 5°C, a 16 L:8D photoperiod, and 30% relative humidity. To generate the *T. ni* larvae used in these experiments, adult moths were provided with a 10% honey water solution as a food source and were allowed to mate inside translucent plastic containers (3.78 L). The mating containers were covered with a sheet of paper towel, on which adult female moths laid their eggs during the night. The paper towels with eggs were collected the next day, surface sterilized for one minute with 5% v/v bleach solution, rinsed with distilled water, air dried, and placed into 10 cm Petri dishes. These dishes were incubated at 25°C for three days to allow for hatching. In all experiments, we used first instar larvae, which advanced to later larval stages during the experiment.

### Effect of Mean Concentrations of Xanthotoxin on *T. ni* Immunity and Caterpillar Weight Gain

We quantified both total hemocyte counts and specific hemocyte types to evaluate cellular immunity. While quantifying individual hemocytes is a more rigorous approach, handling hemocytes for a longer period increases the likelihood of their rupture, making their identification inconclusive. Therefore, combining total hemocyte counts with individual types of hemocytes provides a more reliable and robust method for analysis. Additionally, we assessed humoral immunity by measuring the PO activity as the degree of melanization.

To examine the effect of mean xanthotoxin concentrations on the immune responses of *T. ni* caterpillars, we prepared five artificial diet treatments with xanthotoxin concentrations of 0, 0.5, 1.0, 1.5, or 2 mg/g of diet. These specific concentrations were chosen based on naturally occurring levels of xanthotoxin in two species of Apiaceae (wild parsnip *Pastinaca sativa* and hogweed *Heracleum sphondylium*) (Ode et al. 2004) as well as prior research on the interactions between xanthotoxin and *T. ni* using artificial diets (Lampert et al. 2011; Pearse et al. 2018; Paul et al. 2021). The experimental diets were identical to the standard maintenance diet (Ghosh et al. 2023a, b) except for the addition of xanthotoxin, allowing us to attribute any treatment effects on caterpillar immunity to differences in xanthotoxin concentration. Newly hatched (neonate) *T. ni* caterpillars were individually placed into 44-ml diet cups (Solo^®^) containing one of the five artificial diets. After thirteen days, immunological parameters and body weights were measured on caterpillars from each treatment group. Cellular immunity was assessed by determining the total hemocyte count and the number of four specific hemocyte types (plasmatocytes, granulocytes, oenocytoids, and prohemocytes) by following previously established protocols (Ghosh et al. 2023a, b) (*n* = 11–23). Additionally, we evaluated humoral immunity by measuring PO activity (*n* = 14–24). Briefly, we collected hemolymph by making a lateral incision on each larva’s abdomen using a sterile needle, then transferring the collected hemolymph to pre-chilled Eppendorf tubes. The hemolymph was diluted with phosphate-buffered saline (PBS) at a 1:2 ratio. Approximately 10µL of the diluted hemolymph was placed on a Neubauer hemocytometer, left to settle for 15 min, and then hemocytes were counted under a Leica DM750 microscope at 200x magnification. Hemocyte types were identified morphologically using FITC-tagged phalloidin-DAPI staining (Ghosh and Venkatesan 2019; Fig. S2). For this, 10µL of diluted hemolymph was placed on a pre-chilled Teflon-coated slide, fixed with 4% formaldehyde, washed in PBS, and stained with AlexaFluor 488. Images were captured using a Zeiss LSM-800 confocal microscope, and differential hemocyte counts (DHC) were performed as described by Gupta et al. (1979). To measure PO activity, hemolymph was mixed with ice-cold PBS (1:2 ratio) and centrifuged at 4000 g for 15 min at 4°C. The supernatant was added to a microplate well containing 20µL of PBS and 50µL of 2 mM L-Dopa substrate. The reaction was allowed to proceed for 30 min, and melanization was recorded spectrophotometrically at 490 nm.

### Effect of Variable Plant Toxin Concentrations on Herbivore Immunity

To investigate how fluctuating xanthotoxin concentrations affect immune responses, a separate group of 125 caterpillars was allowed to feed on diet treatments that varied in xanthotoxin levels. These caterpillars were switched every three days between two diets that had a combined average of 1.0 mg xanthotoxin/g of diet. The diet pairs represented three variability conditions: high variability (0 and 2.0 mg xanthotoxin/g diet, *n* = 50), low variability (0.5 and 1.5 mg xanthotoxin/g diet, *n* = 50), and no variability (1.0 and 1.0 mg xanthotoxin/g diet, *n* = 25). The high variability treatment was chosen to simulate extreme fluctuations in toxin levels that herbivores might encounter in nature, testing their ability to cope with rapid changes in exposure levels. The low variability treatment represented moderate fluctuations, allowing us to assess whether immune responses scale proportionally with variability. The no variability treatment served as a control group, ensuring that observed differences could be directly attributed to variability rather than the effects of moving caterpillars between diets. This design ensured that the caterpillars experienced different levels of xanthotoxin variability while maintaining a consistent mean concentration of 1.0 mg/g. To account for potential initial diet effects on the caterpillars’ development, half of the larvae in the low and high variability treatments started on the higher concentration diet, while the other half began on the lower concentration diet. This approach allowed us to isolate the effects of xanthotoxin variability from the initial effects of caterpillar feeding experience on caterpillar immune responses. We measured the same immune parameters as described above.

### Evaluating the Fitness Benefits of Plant Toxin-induced Immunological Changes

To determine whether the immunological changes induced by plant toxins confer actual fitness benefits, such as enhanced resistance to pathogens, we measured the survival of caterpillars challenged with live *Bacillus thuringiensis* (Bt). The Bt culture was initiated from a glycerol stock stored at −80°C. The bacteria were cultured overnight in a nutrient broth at 30°C until they reached an optical density of 0.95 at 600 nm. The culture was then centrifuged, and the resulting pellet was resuspended in 1mL of phosphate-buffered saline (PBS). After thirteen days of feeding on one of the artificial diets, caterpillars from both experiments 1 (five treatments) and 2 (three treatments) were injected with 10µL of PBS carrying a total 10^11^ Bt cells directly into the hemocoel using a nano-injector. As a control, a separate group of larvae was injected with sterile phosphate-buffered saline (0.1 M PBS) to account for any effects of the injection procedure on larval survival. Survival was monitored 36 h post-injection until successful pupation (*n* = 25–40).

### Statistical Analyses

To estimate dose-response functions, we fitted the immunology variables and caterpillar survival in individual Bayesian generalized additive models (GAM; package *brms*, Bürkner 2017). We used a log-linked gamma distribution for caterpillar weight (*n* = 163), a logit-linked beta distribution for PO activity (*n* = 72), a log-linked negative binomial distribution for total hemocyte counts (*n* = 163), a logit-linked multinomial distribution for different hemocyte counts (*n* = 56; plasmatocytes, prohemocytes, oenocytoids, granulocytes), and a logit-linked Bernoulli distribution for caterpillar survival (*n* = 185). We used moderately regularizing priors. We included the xanthotoxin dose as a smoothed predictor using a thin-plate spline with four or five basis dimensions (Wood 2003).

To estimate the mean of each variation treatment group, we fitted Bayesian generalized linear models for caterpillar weight (*n* = 125), PO activity (*n* = 60), total hemocytes count (*n* = 125), hemocyte composition (*n* = 54), and survival (*n* = 105), using the same link function and conditional distribution. We included variation treatment as a predictor. In all models except for survival, we also included the xanthotoxin concentration of the starting diet as a covariate.

### Effect of Nonlinear Averaging

In addition to evaluating the effects of mean toxin levels and variability on herbivore immunity, we computed the effect of variability attributable to nonlinear averaging (i.e., Jensen gap). Herbivore responses to plant defense toxins are likely nonlinear (e.g., Pearse et al. 2018); thus, responses under exposure to variable concentrations of toxins may diverge from those predicted by mean concentrations alone. If the dose-response relationship is convex (i.e., the negative effect of a toxin diminishes at higher levels), nonlinear averaging gives an enhanced response relative to linear averaging. Conversely, if the relationship is concave (i.e., higher toxin levels disproportionately suppress immunity), nonlinear averaging gives a suppressed response relative to linear averaging. Nonlinear averaging therefore provides the response expected under variable toxin exposure based on the shape of the dose response relationship. Comparing this expected response with the observed response allows us to determine whether the effect of toxin variability can be explained by nonlinear averaging alone or whether additional effects of variability are present.

To compute the effect of nonlinear averaging, we performed simulations with the posterior distribution of dose-response functions we estimated. We denoted the dose-response linear predictor function while holding all other variables at the mean as $\eta_d\left(x\right)$. We computed the effect of nonlinear averaging (Jensen gap) as,

1 $$\widehat J=\underbrace{\left\langle g^{-1}\left({\eta\:}_d\left(X\right)\right)\right\rangle}_{Nonlinear\;average}-\underbrace{g^{-1}\left({\eta\:}_d\left(\langle X\rangle\right)\right)}_{Response\:to\;dose\:average}$$

where $g^{-1}(\cdot)$ is the inverse link function, and $\left\langle\cdot\right\rangle$ denotes the mean. The effect was standardized with the pooled standard deviation to facilitate comparison among variables, and can be interpreted akin to Cohen’s *d*.

2 $$SD_{pooled}=\sqrt{\frac{{\sum\:}_i\left(\left\langle Y_i^2\right\rangle-\left\langle Y_i\right\rangle^2\right)\left(n_i-1\right)}{{\sum\:}_i{(n}_i-1)}}$$

Here, $Y_i$ and $n_i$ are the observed response variable and the number of observations belonging to the i^th^ unique treatment group respectively.

To evaluate whether correcting for nonlinear averaging explains the effects of the variation treatments and whether additional factors beyond nonlinear averaging are at play, we needed to compute a new group mean ${\widehat y}_J$ that accounts for the Jensen gap and the experimental effect of switching individual caterpillars between diets. To that end, we added the predicted Jensen gap to the conditional mean of the no variation treatment group via,

3 $$\begin{array}{c}{\widehat y}_J=\\g^{-1}\left(\begin{array}{c}\underbrace{{\eta\:}_n\left(\langle X\rangle\right)}_{No\:variation\;group\:mean}\\+\underbrace{\langle\eta_d\left(X\right)\rangle-\eta_d\left(\langle X\rangle\right)}_{Predicted\:Jensen\:gap}\end{array}\right)\end{array}$$

which correctly restricts the range of $\:{\widehat{y}}_{J}$ with the inverse link function. Pairwise comparisons between the variation treatment group means and Jensen gap corrected group means can then be achieved by subtracting the respective posterior means with each other on the response scale to yield the marginal difference. Differences were considered significant when the 95% credible interval did not include zero. Again, to facilitate comparison, the effects were standardized via the pooled standard deviation. Also, we transformed these differences to percent change and reported that percent change as effect.

Because immunological function is more related to the absolute concentration than the relative concentration of different hemocyte types, we estimated the concentration of different hemocyte types by simply multiplying the mean total hemocyte counts by the mean proportion of each hemocyte type of each treatment group. We used a log link function to evaluate Eqs. 1 and 3. For all calculations, we assume that the total hemocyte count and hemocyte composition are independent within each level of treatment. All calculations were performed in R version 4.3.1 (R Core Team 2023).

## Results

### Fixed Doses of Xanthotoxin Reveal a ‘Window of Enhanced Immunity’ in *T. ni* Caterpillars

**Fig. 1:**
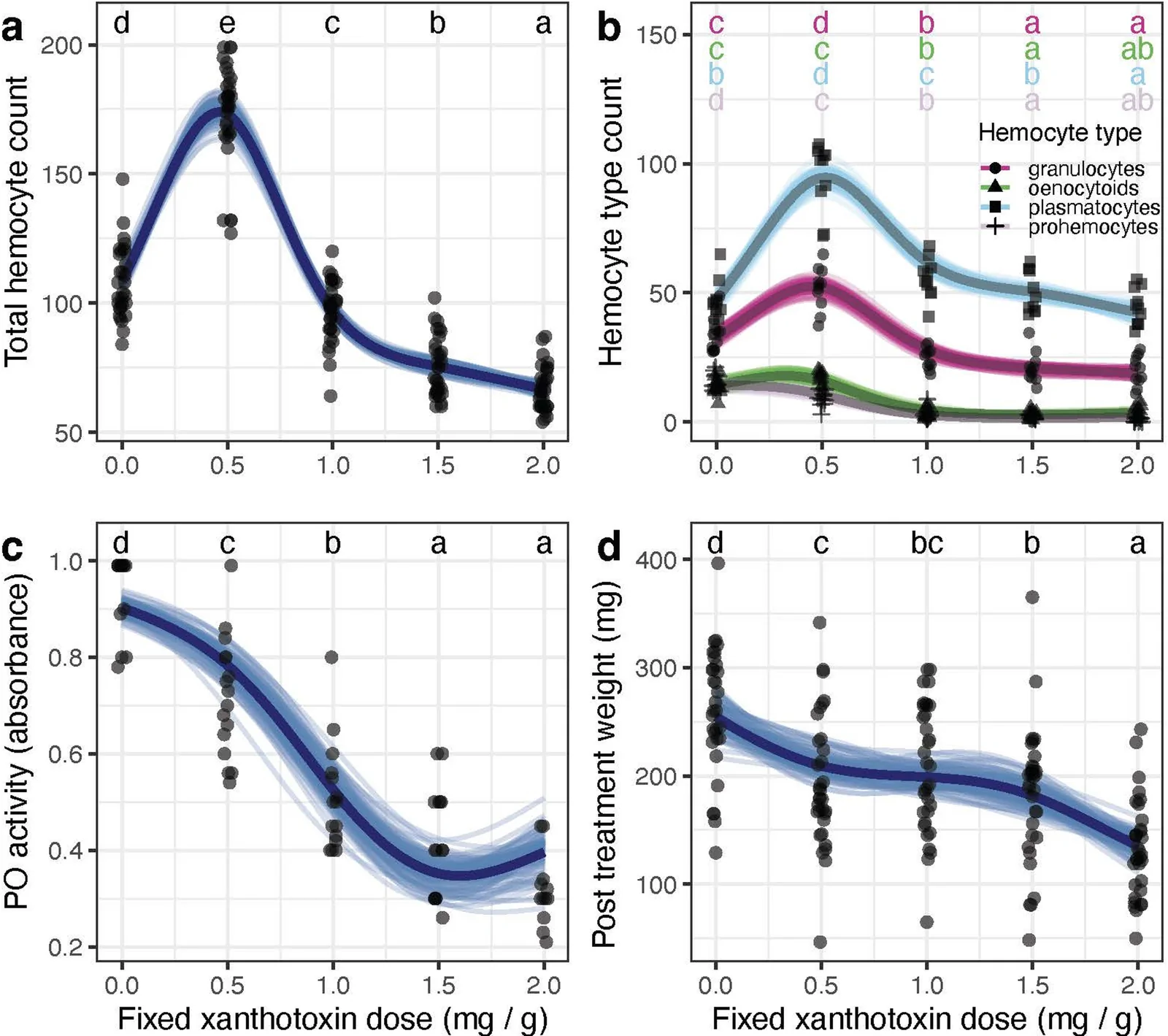
The effect of fixed doses of xanthotoxin on (**a**) total hemocyte count, (**b**) individual hemocyte types in circulation, (**c**) phenol oxidase (PO) activity (a measure of humoral immunity), and (**d**) caterpillar post-treatment weight. Each point represents an individual caterpillar observation. The dark lines depict the mean posterior estimates, while the lighter lines illustrate 100 random draws from the posterior distribution, capturing uncertainty in the model predictions. Different letters (**a**–**d**) indicate statistically significant differences among treatments. In panel b, the letters (**a**–**d**) indicate significant differences among the individual hemocyte types at each fixed xanthotoxin concentration (magenta: granulocytes; green: oenocytoids; blue: plasmatocytes; pink: prohemocytes)

Caterpillars that fed on diets with fixed concentrations of xanthotoxin exhibited significant nonlinear changes in both immunological and weight metrics across the range of xanthotoxin concentrations (Fig. 1, Table S1). Caterpillars consuming 0.5 mg/g xanthotoxin had almost double the number of circulating hemocytes compared to caterpillars feeding on the control diet (0 mg/g) (mean [95% credible intervals]: 0.5 mg/g = 59 [50, 66]%), suggesting the existence of a window of enhanced immunity (Fig. 1a, Table S1). Overall, the different hemocyte types showed broadly similar patterns in relation to xanthotoxin concentration, although the magnitude of the response differed among them, with the strongest response observed for plasmatocytes and weaker responses for oenocytoids and prohemocytes (Fig. 1b, Table S1). Among individual hemocytes, plasmatocytes were the most abundant hemocytes in circulation, followed by granulocytes (Fig. 1b; Figure S1). The number of plasmatocytes was elevated over a broader range of toxin concentrations, with a 5.9–99% increase observed at xanthotoxin doses between 0.5 and 1.5 mg/g (0.5 mg/g = 99 [82, 120]%, 1 mg/g = 30 [18, 43]%, 1.5 mg/g = 5.9 [−3.6, 20]%). In contrast, granulocyte numbers increased by nearly 60% at the 0.5 mg/g dose (Table S1) (59 [38, 82]%). Interestingly, oenocytoid counts were unaffected at xanthotoxin concentrations below 1 mg/g and dropped when dietary concentrations exceeded 1 mg/g (0.5 mg/g = 16 [−8.6, 51] %, 1 mg/g = −67 [−75, −56] %, 1.5 mg/g = −79 [−83, −71]%, 2 mg/g = −75 [−82, −65]%). Prohemocyte counts were suppressed at all xanthotoxin concentrations compared to xanthotoxin free diets (Fig. 1b, Table S1)) (0.5 mg/g = −29 [−44, −8.7]%, 1 mg/g = −82 [−87, −75]%, 1.5 mg/g = −91 [−94, −86]%, 2 mg/g = −86 [−91, −80]%). In contrast, PO activity declined monotonically as xanthotoxin levels increased (by 19–35%) (Fig. 1c), Table S1) (0.5 mg/g = −13 [−18, −7.7]%, 1 mg/g = −42 [−49, −36]%, 1.5 mg/g = −61 [−68, −55]%, 2 mg/g = −56 [−66, −47]%). Caterpillar weight was reduced when they fed on diets with higher xanthotoxin concentrations, particularly at doses of 1.5 mg/g and above, with a 20–30% decrease in body weight in comparison to control (0 mg/g xanthotoxin) (Fig. 1d; Table S1,1.5 mg/g = −28 [−39, −17]%, 2 mg/g = −47 [−54, −38]%).

### Low Variation in Xanthotoxin Exposure can Enhance Immunity

**Fig. 2:**
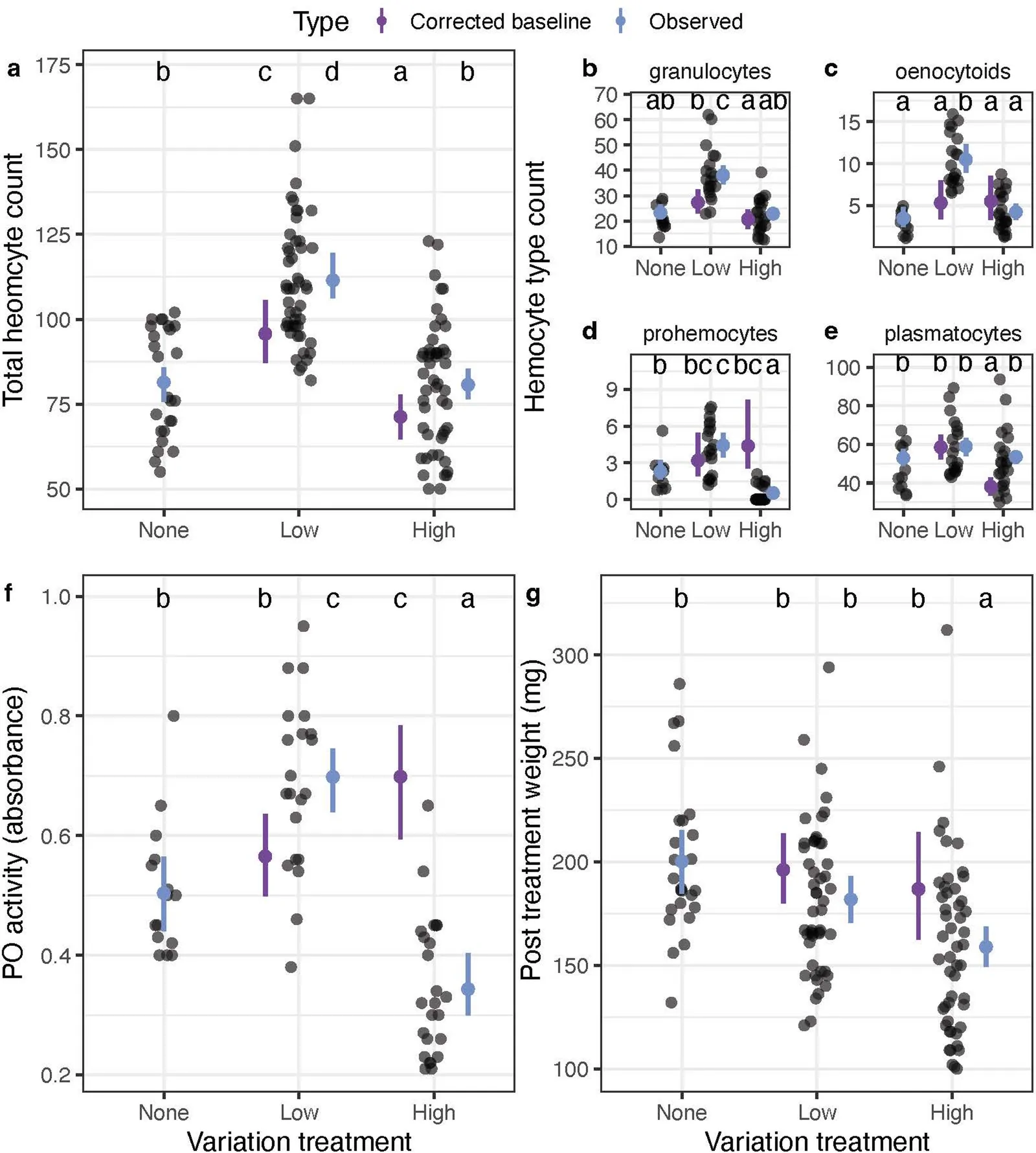
The effect of variable doses of xanthotoxin on (**a**) total hemocyte count, individual hemocyte types in circulation measures as the number of (**b**) granulocytes, (**c**) oenocytoids, (**d**) prohemocytes, (**e**) plasmatocytes, and (**f**) phenol oxidase (PO) activity (a measure of humoral immunity), and (**g**) caterpillar post-treatment weight. Observed (blue) and Jensen gap corrected (purple) caterpillar outcomes are shown as points, lines representing 95% quantiles of the posterior distribution. Each dark point is an observed caterpillar. Variation in xanthotoxin included high variability (0 and 2.0 mg xanthotoxin/g diet), low variability (0.5 and 1.5 mg xanthotoxin/g diet), and no variability (1.0 and 1.0 mg xanthotoxin/g diet). Different letters (**a**–**d**) indicate statistically significant differences among treatments

Caterpillars exposed to the low and high variation treatment groups exhibited distinct immunological and performance responses compared to those in the no variation group (Fig. 2, Table S2-3). Notably, the total number of hemocytes circulating were 28 and 37% higher in the low variation treatment relative to the no variation and high variation treatments respectively (Fig. 2a, Table S2-3). Among individual hemocyte types, granulocytes, plasmatocytes, oenocytoids, and prohemocytes increased in number under low variation conditions compared to no variation conditions (Fig. 2b-e, Table S3). PO activity was 37 and 50% higher in the low variation group compared to either the no variation or the high variation treatments, respectively (Fig. 2f) (GAMs (95% CI): Table S3). These results suggest that the low variation treatment may include toxin concentrations closer to the immunologically optimal concentration, which is likely below the 1 mg/g mean, thereby enhancing immune function more effectively than either the no-variation or high-variation treatments.

We also evaluated whether each trait deviated from linearity by comparing nonlinear versus linear models using ΔLOOIC to predict the accuracy between tested models, where negative values indicate support for nonlinearity. Hemocyte count showed the strongest evidence for a nonlinear response (ΔLOOIC = − 227.1), followed by the proportions of granulocytes, oenocytoids, plasmatocytes, and prohemocytes (ΔLOOIC = − 23.1). Nonlinear models were also significantly favored for weight, PO activity, and survival, although the support was weaker (ΔLOOIC values ranging from − 5.5 to − 26.1).

Overall, the low xanthotoxin variation treatment had immune-enhancing effects in our study, whereas the high variation treatment suppressed the caterpillar’s immune responses. These results suggested that nonlinear averaging alone was not sufficient to explain the effect of toxin variation (Table S2), as correcting for nonlinear averaging did not diminish the deviations but instead accentuated them in many cases.

### Enhanced Immunity Provides Better Survival Against a Pathogen

**Fig. 3:**
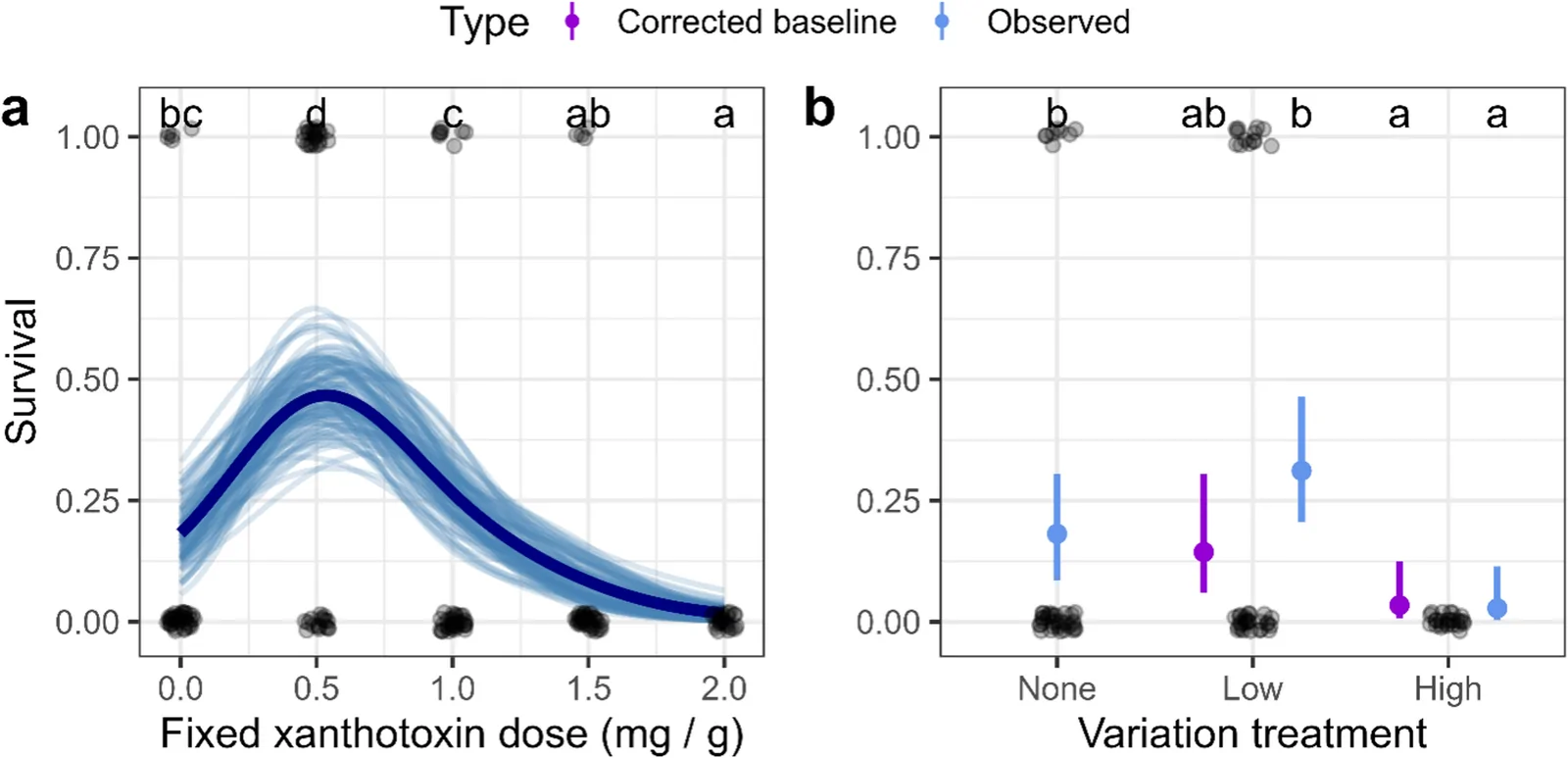
Xanthotoxin mediated enhanced immunity provides better survival against pathogen: Caterpillar survival against *Bacillus thuringiensis* challenge in fixed (**a**) toxin exposure and (**b**) variable toxin exposure treatments. (**a**) The dark line shows the mean posterior distribution, whereas lighter lines show 100 random draws from the posterior distribution. (**b**) Observed (blue) and Jensen gap corrected (purple) caterpillar survival under different toxin variation treatments. Each point is an observed caterpillar. Lines represent 95% quantiles of the posterior distribution. Fixed-dose treatments included xanthotoxin concentration of 0, 0.5, 1.0, 1.5, or 2 mg/g of diet; Variability-dose included high variability (0 and 2.0 mg xanthotoxin/g diet), low variability (0.5 and 1.5 mg xanthotoxin/g diet), and no variability (1.0 and 1.0 mg xanthotoxin/g diet). Different letters (a–d) indicate statistically significant differences among treatments

We found that plant toxin-mediated immunological changes conferred enhanced defense against pathogens (Fig. 3a-b). In the absence of xanthotoxin, caterpillars exhibited only a 18% ([8.5, 31]%) survival rate against *Bacillus thuringiensis* (Bt) infection. However, exposure to a mean dose of 0.5 mg/g xanthotoxin significantly increased their survival to 47% ([33, 61]%) before dropping to 27% ([16,39]%) at 1.0 mg/g, 8.3% ([3.0,16]%) at 1.5 mg/g and 1.7% at 2 mg/g ([0.23, 5.3]%) (Fig. 3a, Table S1). We found no significant difference in survival when infected with *Bacillus thuringiensis* (Bt) between the two control groups: PBS-injected and no-xanthotoxin (Fig. S2). Caterpillars in the low variation treatment group showed a 32% ([19, 45]%) survival rate, while the high variation group had almost no survivors (4.0 [0.040, 14%]). The no variability treatment group exhibited a 19% ([9.7, 31]%) survival rate (Fig. 3b, Table S3). Overall, under our experimental conditions, caterpillars fed a diet with low variation in xanthotoxin concentration experienced improved defenses against pathogens (Table S3). The change in survival due to low or high variation treatments was fully explained by nonlinear averaging alone (Table S2). Interestingly, in our cross-treatment analysis (Table S4), caterpillar survival was strongly positively correlated with total hemocyte count (Spearman *r* = 0.88, *P =* 0.0040, *n* = 8), confirming its role in pathogen immunity.

## Discussion

Our work shows that a single plant toxin can both enhance and suppress the immune response of an herbivorous insect, depending on the dose. That low to moderate levels of xanthotoxin enhanced the immune function of *T. ni*, while higher concentrations suppressed it is a clear example of the hormetic effects of a plant toxin on herbivore immune function. Furthermore, the relationship between *T. ni* survivorship after infection with Bt and xanthotoxin concentration consumed is also hormetic with peak survival occurring at the same toxin concentration that maximizes *T. ni* immune function. Our study provides a link between plant chemistry and herbivore immune responses to pathogen challenge. Variation in defensive chemistry among host plants could therefore alter herbivore susceptibility to pathogens, potentially influencing pathogen driven mortality and, consequently, the severity of insect outbreaks (Elderd et al. 2013).

There are at least two potential scenarios that could explain a biphasic relationship between plant toxin concentrations and herbivore immunocompetence. First, plant toxins may *directly* enhance herbivore immunity at lower concentrations before having a suppressive effect at higher concentrations, similar to the hormetic effects of pesticides on other aspects of insect fitness (Cutler et al. 2022). Although the underlying mechanisms are unclear, studies in model organisms, including *Caenorhabditis elegans*, *Drosophila melanogaster*, and humans, suggest that transcriptional networks are upregulated, enhancing physiological function when exposed to mild doses of stressors (Calabrese 2005; Calabrese and Blain 2009). While the structure of these networks may differ among organisms and stress types, a biphasic response to the level of stressor is a hallmark. Reactive oxygen species (ROS) likely play a central role in this stress response as suggested by their involvement in almost all physiological stress conditions (Bauer et al. 2024). For example, pathogen exposure induces ROS production, a response that can also be elicited by pathogen-associated molecular patterns (PAMPs) (Molina-Cruz et al. 2008). At low levels, ROS promote anabolic processes that support immune cell proliferation and boost resistance to infection. However, when ROS production exceeds the capacity of antioxidant defenses, cellular damage becomes the dominant outcome (Monaghan et al. 2009). Since this stress response is broadly conserved, we predict that a qualitatively similar biphasic relationship could occur in more closely coevolved plant–herbivore systems, although the dose at which immune stimulation shifts to suppression would likely differ among herbivores (Ode and Ghosh 2025).

In our study, one key distinction is that hemocyte numbers increased while PO activity declined at low to intermediate xanthotoxin doses. At these toxin doses, oxidative signals may transiently promote cellular immune proliferation but suppress the proPO/PO cascade owing to strong pleiotropic constraints and high metabolic cost, particularly given the role of the proPO/PO cascade in molting, wound repair, and tissue differentiation (Lu et al. 2014; Arakane et al. 2005; Lavine and Strand 2002). Thus, toxin exposure may induce cellular immunity while downregulating PO to preserve developmental homeostasis. Whether insect herbivores such as *T. ni* have evolved specialized transcriptional networks that both mitigate diverse stressors and selectively activate immune pathways remains an open question.

Second, perceived threat of parasitism or pathogen attack may serve as a cue that influences host plant choice by ovipositing herbivore females, favoring plants with higher toxin concentrations that could increase offspring survival following attack. Such an association could arise if ovipositing females alter host plant choice in response to natural enemy pressure, preferentially placing offspring on plants with higher concentrations of compounds that provide protection against parasites or pathogens (Ghosh et al. 2022). Indeed, maternal dietary or oviposition choices that increase offspring exposure to antiparasitic plant compounds have been described as a form of transgenerational medication (Singer et al. 2014). Under such conditions, plant toxin exposure could become associated with natural enemy risk, potentially favoring corresponding adjustments in offspring immune investment. Herbivores feeding on plants with low toxin concentrations likely accumulate or sequester toxins at levels insufficient to provide significant protection from parasites or pathogens, so herbivores would benefit by increasing investment in immunity. At higher levels of toxin consumption, accumulated or sequestered toxins within the herbivore may begin to protect against parasites as well as necessitate investment in detoxification (especially for non-sequestering species), both of which subsequently trade off with investment in immunity. Several examples of induced prophylaxis exist including the collection of spruce resin by wood ants to prevent attack by bacterial and fungal pathogens (Christe et al. 2003; Castella et al. 2008) and the expression of enhanced immunity by caterpillars under crowded conditions which raises the risk of the spread of disease (*Spodoptera littoralis* – Cotter et al. 2004; *Lymantria dispar* – Reilly and Hajek 2008; *Plodia interpunctella* – Triggs and Knell 2012).

Moreover, variance in toxin exposure clearly affects the immune response of *T. ni*. In our study we found that caterpillars fed a low variance diet (containing 0.5 and 1.5 mg/g xanthotoxin) outperformed caterpillars fed a no variance (1.0 mg/g xanthotoxin) or high variance (0 and 2.0 mg/g xanthotoxin) diet both in terms of cellular immune response and survivorship when infected with Bt. Rather than responding to the mean toxin level (1.0 mg/g xanthotoxin), caterpillars in the variance treatments appear to be more strongly affected by the specific dietary toxin concentrations encountered. That the low variance treatment included a toxin concentration (0.5 mg/g xanthotoxin) that is well within the window of enhanced immunity may account for the increased immunity and survivorship of caterpillars in this treatment compared to the other two treatments. The other two variance treatments include concentrations either at the edge (0 mg/g or 1.0 mg/g xanthotoxin) or outside (2.0 mg/g xanthotoxin) the window of enhanced immunity. Had we picked a different mean toxin value to center our variation treatments around (i.e., 0.5 mg/g), we would have expected that the no variation treatment would outperform both the low and high variation treatments. Moreover, several larval traits deviated from the nonlinear averaging projection for reasons that cannot yet be resolved without explicit mathematical modeling, inviting future work to identify and test these hidden physiological drivers.

Nevertheless, caterpillar survival and individual immune traits (like PO activity) differed in how they responded to toxin variation. Whereas survival followed the expectations of nonlinear averaging, immune responses did not. This result suggests that additional factors beyond nonlinear averaging may be driving the effect of toxin variation on immunity. For example, a time lag between the new levels of toxin a caterpillar encounters and the caterpillar’s ability to match its detoxification capacity can create periods of suboptimal detoxification activity. Indeed, upregulation of detoxifying P450 enzymes in *T. ni* can take as long as 12 h, during which the toxin can do significant damage (Fang et al. 2007). Conversely, limiting resources that are otherwise used for pathogen immunity may be used to maintain unneeded enzymes at periods of low toxin exposure (Vogel et al. 2014). While the importance of these mechanisms remains to be tested, our results offer insight into how variation in plant toxins may affect herbivore responses and the outcomes of trophic interactions in heterogeneous environments.

Ultimately, our findings indicate that dose-response relationships of herbivore immunity follow a hormetic pattern. Although this pattern has not been demonstrated previously, it likely generalizes to many plant-herbivore systems. For example, monarchs feeding on high-cardenolide host plants show reduced survival and weaker immune responses, illustrating the same trade-off between toxin processing and defense investment that we observed (Sternberg et al. 2012). When cardenolide levels fluctuate widely, monarchs struggle to maintain stable detoxification and immune function, suggesting that rapid shifts in toxin exposure can undermine their capacity to mount effective immune responses (Tan et al. 2019). Similarly, the tobacco hornworm (*Manduca sexta*), which feeds on plants in the family Solanaceae, can encounter variable levels of nicotine across different host plants and even among tissues within a single plant. Moderate exposure to nicotine can enhance resistance to pathogens by stimulating immune pathways, such as the production of antimicrobial peptides (Garvey et al. 2021). Together these examples show that variable toxin exposure is a normal feature of herbivore–plant interactions and that some degree of fluctuation can be accommodated or even leveraged for improved immunity. However, too much variation might overwhelm physiological plasticity, leading to negative outcomes. Future studies could help define these thresholds and clarify the underlying mechanisms.

## Supplementary Information

Below is the link to the electronic supplementary material.

Supplementary Material 1 (DOCX 622 KB)

## Data Availability

The raw data generated in this study are available on Dryad (https://doi.org/10.5061/dryad.prr4xgxx2). A cloned code repository used for this study is available on Zenodo (https://doi.org/10.5281/zenodo.14207453). All materials are currently accessible via a private peer-review link (http://datadryad.org/stash/share/TClbEbEEGHAf5ftiv9WjwtGiTpETzSMsohA8z4RsRzs).

## References

[CR1] Arakane Y, Muthukrishnan S, Beeman RW, Kanost MR, Kramer KJ (2005) Laccase 2 is the phenoloxidase gene required for beetle cuticle tanning. Proc Natl Acad Sci U S A 102(32):11337–11342. 10.1073/pnas.0504982102 PMC1183588 16076951

[CR2] Bauer M, Ermolaeva M, Singer M, Wetzker R, Soares MP (2024) Hormesis as an adaptive response to infection. Trends Mol Med 30(7):633–641. 10.1016/j.molmed.2024.04.012 38744580

[CR3] Beran F, Petschenka G (2022) Sequestration of plant defense compounds by insects. Ann Rev Entomol 67:163–183. 10.1146/annurev-ento-062821-062319 34995091

[CR4] Berenbaum M (1981) Patterns of Furanocoumarin Distribution and Insect Herbivory in the Umbelliferae: Plant Chemistry and Community Structure. Ecology 62:1254–1266. 10.2307/1937290

[CR5] Berry R 3rd, López-Martínez G (2020) A dose of experimental hormesis: When mild stress protects and improves animal performance. Comp Biochem Physiol A Mol Integr Physiol 242(110658):110658. 10.1016/j.cbpa.2020.110658 PMC7066548 31954863

[CR6] Bürkner P-C (2017) brms: An R Package for Bayesian Multilevel Models Using Stan. J Stat Softw 80(1):1–28. 10.18637/jss.v080.i01

[CR7] Calabrese EJ (2005) Hormetic dose-response relationships in immunology: occurrence, quantitative features of the dose response, mechanistic foundations, and clinical implications. Crit Rev Toxicol 35(2–3):89–295. 10.1080/10408440590917044 15839378

[CR8] Calabrese EJ (2014) Hormesis: a fundamental concept in biology, toxicology and pharmacology. Annu Rev Pharmacol Toxicol 54:1–27

[CR9] Calabrese EJ, Baldwin LA (2003) Hormesis: the dose response revolution. Annu Rev Pharmacol Toxicol 43:175–197 10.1146/annurev.pharmtox.43.100901.140223 12195028

[CR10] Calabrese EJ, Blain RB (2009) Hormesis and plant biology. Environmental Pollution (Barking, Essex: 1987) 157(1):42–48. 10.1016/j.envpol.2008.07.028 18790554

[CR11] Castella G, Chapuisat M, Christe P (2008) Prophylaxis with resin in wood ants. Anim Behav 75:1591–1596. 10.1016/j.anbehav.2007.10.014

[CR12] Christe P, Oppliger A, Bancalà F, Castella G, Chapuisat M (2003) Evidence for collective medication in ants. Ecol Lett 6(1):19–22. 10.1046/j.1461-0248.2003.00395.x

[CR13] Cotter SC, Hails RS, Cory JS, Wilson K (2004) Density dependent prophylaxis and condition dependent immune function in lepidopteran larvae. J Anim Ecol 73(2):283–293. 10.1111/j.00218790.2004.00806.x

[CR14] Cutler GC, Amichot M, Benelli G, Guedes RNC, Qu Y, Rix RR, Ullah F, Desneux N (2022) Hormesis and insects: Effects and interactions in agroecosystems. Sci Total Environ 825(153899):153899. 10.1016/j.scitotenv.2022.153899 35181361

[CR15] Elderd BD, Rehill BJ, Haynes KJ, Dwyer G (2013) Induced plant defenses, host-pathogen interactions, and forest insect outbreaks. Proc Natl Acad Sci USA 110(37):14978–14983. 10.1073/pnas.1300759110 PMC3773759 23966566

[CR16] Fang XK, Huang DF, Wang ZX, Wan CL, Sun T, Xu WJ, Liu CY, Zhou P, Qiao ZD (2007) Identification of the proteins related to cytochrome P450 induced by fenvalerate in a *Trichoplusia ni* cell line. Cell Biol Toxicol 23(6):445–57. 10.1007/s10565-007-9006-1 17484068

[CR17] Garvey M, Bredlau J, Kester K, Creighton C, Kaplan I (2021) Toxin or medication? Immunotherapeutic effects of nicotine on a specialist caterpillar. Funct Ecol 35(3):614–626. 10.1111/1365-2435.13743

[CR18] Ghosh E, Paul RL, Ode PJ (2023a) Plant species with higher chemical defences enhance herbivore cellular immunity with differential effectiveness against two parasitoid species. Funct Ecol 37(5):1492–1503. 10.1111/1365-2435.14292

[CR19] Ghosh E, Sasidharan A, Ode PJ, Venkatesan R (2022) Oviposition Preference and Performance of a Specialist Herbivore Is Modulated by Natural Enemies, Larval Odors, and Immune Status. J Chem Ecol 48(7–8):670–682. 10.1007/s10886-022-01363-5 35604580

[CR20] Ghosh E, Tafesh-Edwards GSY, Eleftherianos I, Goldin SL, Ode PJ (2023b) The plant toxin 4-methylsulfinylbutyl isothiocyanate decreases herbivore performance and modulates cellular and humoral immunity. PLoS One 18(8):e0289205. 10.1371/journal.pone.0289205 PMC10395821 37531339

[CR21] Ghosh E, Venkatesan R (2019) Plant volatiles modulate immune responses of Spodoptera litura. J Chem Ecol 45(8):715–724 10.1007/s10886-019-01091-3 31385154

[CR22] Groen SC, Whiteman NK (2022) Ecology and Evolution of Secondary Compound Detoxification Systems in Caterpillars. In: Marquis RJ, Koptur S (eds) Caterpillars in the Middle: Tritrophic Interactions in a Changing World. Springer International Publishing, pp 115–163. 10.1007/978-3-030-86688-4_5

[CR23] Gupta AP (1979) Haemocyte types: their structure, synonymies, interrelationships and taxonomic significance. In: Gupta AP (ed) Insect Haemocytes. Cambridge University Press, pp 85–127

[CR24] Hillyer JF (2016) Insect immunology and hematopoiesis. Dev Comp Immunol 58:102–118. 10.1016/j.dci.2015.12.006 PMC4775421 26695127

[CR25] Holmes KD, Blubaugh CK (2023) A guide to 23 global syntheses of plant diversity effects: Unpacking consensus and incongruence across trophic levels. Q Rev Biol 98(3):121–148. 10.1086/726687

[CR26] Lampert EC, Zangerl AR, Berenbaum MR, Ode PJ (2011) Generalist and specialist host-parasitoid associations respond differently to wild parsnip (Pastinaca sativa) defensive chemistry. Ecol Entomol 36:52–61

[CR27] Lavine MD, Strand MR (2002) Insect hemocytes and their role in immunity. Insect Biochem Mol Biol 32(10):1295–309. 10.1016/s0965-1748(02)00092-9 12225920

[CR28] Lu A, Zhang Q, Zhang J, Yang B, Wu K, Xie W, Luan Y-X, Ling E (2014) Insect prophenoloxidase: the view beyond immunity. Front Physiol 5:252. 10.3389/fphys.2014.00252 PMC4092376 25071597

[CR29] Mattson MP (2008) Hormesis defined. Ageing Res Rev 7(1):1–7. 10.1016/j.arr.2007.08.007 PMC2248601 18162444

[CR30] Molina-Cruz A, DeJong RJ, Charles B, Gupta L, Kumar S, Jaramillo-Gutierrez G, Barillas-Mury C (2008) Reactive oxygen species modulate *Anopheles gambiae* immunity against bacteria and *Plasmodium*. J Biol Chem 283(6):3217–3223. 10.1074/jbc.M705873200 18065421

[CR31] Monaghan P, Metcalfe NB, Torres R (2009) Oxidative stress as a mediator of life history trade-offs: mechanisms, measurements and interpretation. Ecol Lett 12(1):75–92. 10.1111/j.1461-0248.2008.01258.x 19016828

[CR32] Nishida R (2002) Sequestration of defensive substances from plants by Lepidoptera. Ann Rev Entomol 47:57–92 10.1146/annurev.ento.47.091201.145121 11729069

[CR33] Ode PJ, Berenbaum MR, Zangerl AR, Hardy ICW (2004) Host plant, host plant chemistry and the polyembryonic parasitoid *Copidosoma sosares*: indirect effects in a tritrophic interaction. Oikos 104:388–400

[CR34] Ode PJ, Ghosh E (2025) The dose makes the poison: plant toxin concentrations and herbivore immunity against pathogens and parasitoids. Curr Opin Insect Sci 71:101405. 10.1016/j.cois.2025.101405 40578433

[CR35] Pan VS, Ghosh E, Ode PJ, Wetzel WC, Gilbert KJ, Pearse IS (2025a) Large differences in herbivore performance emerge from simple herbivore behaviours and fine-scale spatial heterogeneity in phytochemistry. Ecol Lett 28:e70044. 10.1111/ele.70044 PMC11687352 39737776

[CR36] Pan VS, Gilbert KJ, Wetzel WC (2025b) Mean plant toxicity modulates the effects of plant defense variability. Ecology 106(2):e70012. 10.1002/ecy.70012 PMC11792111 39902654

[CR37] Paul RL, Pearse IS, Ode PJ (2021) Fine-scale plant defence variability increases top‐down control of an herbivore. Funct Ecol 35(7):1437–1447. 10.1111/1365-2435.13808

[CR38] Pearse IS, Paul R, Ode PJ (2018) Variation in plant defense suppresses herbivore performance. Curr Biology: CB 28(12):1981–1986e. 10.1016/j.cub.2018.04.070 29887306

[CR39] Quintero C, Lampert EC, Bowers MD (2014) Time is of the essence: direct and indirect effects of plant ontogenetic trajectories on higher trophic levels. Ecology 95(9):2589–2602. 10.1890/13-2249.1

[CR40] R Core Team (2023) R: A language and environment for statistical computing. R Foundation for Statistical Computing. https://www.r-project.org/

[CR41] Reilly JR, Hajek AE (2008) Density-dependent resistance of the gypsy moth *Lymantria dispar* to its nucleopolyhedrovirus, and the consequences for population dynamics. Oecologia 154(4):691–701. 10.1007/s00442-007-0871-3 17968593

[CR42] Reudler JH, Biere A, Harvey JA, Nouhuys SV (2011) Differential performance of a specialist and two generalist herbivores and their parasitoids on Plantago lanceolata. J Chem Ecol 37:765–778 10.1007/s10886-011-9983-7 PMC3125503 21691810

[CR43] Ruhnke H, Schädler M, Klotz S, Matthies D, Brandl R (2009) Variability in leaf traits, insect herbivory and herbivore performance within and among individuals of four broad-leaved tree species. Basic Appl Ecol 10(8):726–736. 10.1016/j.baae.2009.06.006

[CR44] Shikano I (2017) Evolutionary Ecology of Multitrophic Interactions between Plants, Insect Herbivores and Entomopathogens. J Chem Ecol 43(6):586–598. 10.1007/s10886-017-0850-z 28526946

[CR45] Singer MS, Lichter-Marck IH, Farkas TE, Aaron E, Whitney KD, Mooney KA (2014) Herbivore diet breadth mediates the cascading effects of carnivores in food webs. Proc Natl Acad Sci U S A 111(26):9521–6. 10.1073/pnas.1401949111 PMC4084428 24979778

[CR46] Singer MS, Mace KC, Bernays EA (2009) Self-medication as adaptive plasticity: increased ingestion of plant toxins by parasitized caterpillars. PLoS One 4(3):e4796. 10.1371/journal.pone.0004796 PMC2652102 19274098

[CR47] Slikker W, Jr, Andersen ME, Bogdanffy MS, Bus JS, Cohen SD, Conolly RB, David RM, Doerrer NG, Dorman DC, Gaylor DW, Hattis D, Rogers JM, Setzer RW, Swenberg JA, Wallace K (2004) Dose-dependent transitions in mechanisms of toxicity. Toxicol Appl Pharmcol 201(3):203–225. 10.1016/j.taap.2004.06.019 15582645

[CR48] Smilanich AM, Dyer LA, Chambers JQ, Bowers MD (2009) Immunological cost of chemical defence and the evolution of herbivore diet breadth. Ecol Lett 12(7):612–621. 10.1111/j.1461-0248.2009.01309.x 19392713

[CR49] Smilanich AM, Muchoney ND (2022) Host Plant Effects on the Caterpillar Immune Response. In: Marquis RJ, Koptur S (eds) Caterpillars in the Middle: Tritrophic Interactions in a Changing World. Springer International Publishing, pp 449–484. 10.1007/978-3-030-86688-4_14

[CR50] Sternberg ED, Lefèvre T, Li J, de Castillejo CLF, Li H, Hunter MD, de Roode JC (2012) Food plant derived disease tolerance and resistance in a natural butterfly-plant-parasite interactions. Evolution 66(11):3367–3376. 10.1111/j.1558-5646.2012.01693.x 23106703

[CR51] Sutherland DWS, Greene GL (1984) *Cultivated wild host plants. Suppression management cabbage looper populations* (pp. 1–13)

[CR52] Tan W-H, Acevedo T, Harris EV et al (2019) Transcriptomics of monarch butterflies (Danaus plexippus) reveals that toxic host plants alter expression of detoxification genes and down-regulate a small number of immune genes. Mol Ecol 28:4845–4863. 10.1111/mec.15219 31483077

[CR53] Triggs AM, Knell RJ (2012) Parental diet has strong transgenerational effects on offspring immunity. Funct Ecol 26:1409–1417. 10.1111/j.1365-2435.2012.02051.x

[CR54] Vogel H, Musser RO, Celorio-Mancera P (2014) M.d.l. Transcriptome Responses in Herbivorous Insects Towards Host Plant and Toxin Feeding. In Annual Plant Reviews (eds C. Voelckel and G. Jander). 10.1002/9781118829783.ch6

[CR55] Wan Y, Liu J, Mai Y, Hong Y, Jia Z, Tian G, Liu Y, Liang H, Liu J (2024) Current advances and future trends of hormesis in disease. Npj Aging 10(1):26. 10.1038/s41514-024-00155-3 PMC11096327 38750132

[CR57] Wetzel WC, Kharouba HM, Robinson M, Holyoak M, Karban R (2016) Variability in plant nutrients reduces insect herbivore performance. Nature 539(7629):425–427. 10.1038/nature20140 27749815

[CR58] Wood SN (2003) Thin plate regression splines. J Royal Stat Society: Ser B (Statistical Methodology) 65(1):95–114. 10.1111/1467-9868.00374

[CR59] Zangerl AR, Berenbaum MR (1994) Spatial, temporal, and environmental limits on xanthotoxin induction in wild parsnip foliage. Chemoecology 5:37–42. 10.1007/BF01259971

