# Supplementary material for: Herbivore Immunity Exhibits a Biphasic Relationship with Plant Defense Toxin Concentrations
Source: J Chem Ecol. 2026 Oct 13;52(5):95. doi: 10.1007/s10886-026-01772-w (PMC13658300; doi:10.1007/s10886-026-01772-w)
Supplement: Supplementary file 1 [file 10886_2026_1772_MOESM1_ESM.docx]

**Plant defense toxins exhibit a biphasic relationship with herbivore immunity**

Enakshi Ghosh, Vincent S. Pan, Ian Pearse, Paul J. Ode

**Table S1.** Pairwise contrasts between groups that received a non-zero fixed dose of xanthotoxin and the control group that received no xanthotoxin (mean [95% credible intervals]). % change calculated as ${(\langle y}_{\mathrm{candidate}}\rangle-\langle y_{\mathrm{null}}\rangle)/{\langle y}_{\mathrm{null}}\rangle\cdot100$, where the group means are back transformed to the response scale. Significant effects are bolded.

| **Variable** | **Fixed dose (mg/g)** | **% change from control** |
| --- | --- | --- |
| Weight (mg) | 0.5 | **-17 [-28, -6]** |
| Weight (mg) | 1 | **-21 [-29, -11]** |
| Weight (mg) | 1.5 | **-28 [-39, -17]** |
| Weight (mg) | 2 | **-47 [-54, -38]** |
| PO activity (proportion) | 0.5 | **-13 [-18, -7.7]** |
| PO activity (proportion) | 1 | **-42 [-49, -36]** |
| PO activity (proportion) | 1.5 | **-61 [-68, -55]** |
| PO activity (proportion) | 2 | **-56 [-66, -47]** |
| Total hemocyte count | 0.5 | **59 [50, 66]** |
| Total hemocyte count | 1 | **-11 [-16, -5.6]** |
| Total hemocyte count | 1.5 | **-31 [-35, -25]** |
| Total hemocyte count | 2 | **-39 [-43, -35]** |
| Granulocyte count | 0.5 | **59 [38, 82]** |
| Granulocyte count | 1 | **-14 [-24, -0.29]** |
| Granulocyte count | 1.5 | **-36 [-44, -28]** |
| Granulocyte count | 2 | **-42 [-51, -33]** |
| Oenocytoid count | 0.5 | 16 [-8.6, 51] |
| Oenocytoid count | 1 | **-67 [-75, -56]** |
| Oenocytoid count | 1.5 | **-79 [-83, -71]** |
| Oenocytoid count | 2 | **-75 [-82, -65]** |
| Plasmatocyte count | 0.5 | **99 [82, 120]** |
| Plasmatocyte count | 1 | **30 [18, 43]** |
| Plasmatocyte count | 1.5 | 5.9 [-3.6, 20] |
| Plasmatocyte count | 2 | **-11 [-20, -0.73]** |
| Prohemocyte count | 0.5 | **-29 [-44, -8.7]** |
| Prohemocyte count | 1 | **-82 [-87, -75]** |
| Prohemocyte count | 1.5 | **-91 [-94, -86]** |
| Prohemocyte count | 2 | **-86 [-91, -80]** |
| Survival | 0.5 | **210 [45, 450]** |
| Survival | 1 | 73 [-28, 240] |
| Survival | 1.5 | -46 [-86, 42] |
| Survival | 2 | **-89 [-99, -58]** |

**Table S2.** Comparison between the expected and observed effect of variation treatment for different caterpillar immunological and performance outcomes. Significant effects are bolded. Effects are standardized by the pooled standard deviation (mean [95% credible intervals]). ‘Jensen gap’ shows the predicted effect of nonlinear averaging derived from eqn. 1 and 2. ‘Deviation from no variation’ shows the observed contrast between the variation treatment group relative to the no variation treatment group. ‘Deviation from expectation’ shows the contrast between the variation treatment group relative to the Jensen gap corrected no variation treatment group (eqn. 3).

| **Variable** | **Variance**  **treatment** | **Jensen gap (*SD*)** | **Deviation from no variation (*SD*)** | **Deviation from expectation (*SD*)** |
| --- | --- | --- | --- | --- |
| Weight | Low | -0.060 [-0.19, 0.058] | -0.48 [-1.0, 0.032] | -0.38 [-0.94, 0.16] |
|  | High | -0.068 [-0.48, 0.34] | **-1.1 [-1.7, -0.64]** | **-0.78 [-1.6, -0.059]** |
| PO activity | Low | **0.40 [0.094, 0.70]** | **1.5 [0.79, 2.2]** | **1.0 [0.30, 1.8]** |
|  | High | **1.2 [0.45, 1.9]** | **-1.3 [-2.0, -0.63]** | **-2.8 [-3.7, -1.9]** |
| Granulocyte  count | Low | **1.5 [0.98, 2.1]** | **2.0 [1.4, 2.7]** | **1.5 [0.65, 2.2]** |
|  | High | -0.42 [-1.1, 0.28] | -0.099 [-0.66, 0.45] | 0.28 [-0.42, 0.9] |
| Oenocytoid count | Low | **1.2 [0.82, 1.6]** | **1.4 [0.95, 1.8]** | **1.0 [0.41, 1.6]** |
|  | High | **1.1 [0.59, 1.6]** | 0.15 [-0.15, 0.44] | -0.26 [-0.88, 0.23] |
| Plasmatocyte count | Low | **1.9 [1.1, 2.7]** | **0.86 [0.0037, 1.7]** | 0.056 [-1.0, 1.1] |
|  | High | **-3.1 [-4.0, -2.2]** | 0.12 [-0.73, 0.93] | **2.0 [1.2, 2.8]** |
| Prohemocyte  count | Low | **0.86 [0.54, 1.2]** | **0.42 [0.12, 0.73]** | 0.22 [-0.25, 0.62] |
|  | High | **1.4 [0.97, 1.9]** | **-0.36 [-0.58, -0.18]** | **-0.83 [-1.5, -0.39]** |
| Survival | Low | 0.023 [-0.27, 0.32] | 0.34 [-0.12, 0.80] | 0.43 [-0.072, 0.89] |
|  | High | **-0.45 [-0.83, -0.10]** | **-0.39 [-0.73, -0.086]** | -0.0014 [-0.21, 0.23] |

**Table S3.** Pairwise contrasts between variation treatment groups (mean [95% credible intervals]). % change calculated as ${(\langle y}_{\mathrm{candidate}}\rangle-\langle y_{\mathrm{null}}\rangle)/{\langle y}_{\mathrm{null}}\rangle\cdot100$, where the group means are back transformed to the response scale. Significant effects are bolded.

| **Variable** | **Contrasts** | **Marginal effect (% change)** |
| --- | --- | --- |
| Weight (mg) | Low var. – No var. | -8.9 [-18, 0.64] |
|  | High var. – No var. | **-21 [-29, -13]** |
|  | High var. – Low var. | **-13 [-20, -5.8]** |
| PO activity (proportion) | Low var. – No var. | **37 [17, 60]** |
|  | High var. – No var. | **-31 [-44, -17]** |
|  | High var. – Low var. | **-50 [-58, -41]** |
| Granulocyte count | Low var. – No var. | **63 [39, 92]** |
|  | High var. – No var. | -2.6 [-18, 16] |
|  | High var. – Low var. | **-40 [-48, -31]** |
| Oenocytoids count | Low var. – No var. | **210 [110, 350]** |
|  | High var. – No var. | 25 [-18, 85] |
|  | High var. – Low var. | **-59 [-70, -46]** |
| Plasmatocytes count | Low var. – No var. | **13 [0.05, 27]** |
|  | High var. – No var. | 1.9 [-9.7, 14] |
|  | High var. – Low var. | -9.4 [-18, 0.23] |
| Prohemocytes count | Low var. – No var. | **99 [19, 220]** |
|  | High var. – No var. | **-76 [-90, -53]** |
|  | High var. – Low var. | **-88 [-95, -77]** |
| Survival (proportion) | Low var. – No var. | 87 [-17, 280] |
|  | High var. – No var. | **-78 [-98, -25]** |
|  | High var. – Low var. | **-87 [-99, -58]** |

**Table S4**. Pairwise correlation between average survival and average immune function or average performance of treatment groups. Significant correlations are bolded.

| **Variable** | **Spearman correlation** | ***P*** | ***n*** |
| --- | --- | --- | --- |
| Plasmatocyte count | **0.71** | **0.048** | 8 |
| Prohemocyte count | **0.75** | **0.033** | 8 |
| Oenocytoids count | 0.39 | 0.35 | 8 |
| Granulocytes count | **0.72** | **0.043** | 8 |
| Weight | 0.46 | 0.25 | 8 |
| Total hemocyte count | **0.88** | **0.0040** | 8 |
| PO absorbance | **0.75** | **0.032** | 8 |

**Figure S1**: Effect of fixed doses of xanthotoxin on hemocytes proportional composition. Dose-response functions were estimated using a Bayesian generalized additive model with a logit-linked multinomial distribution. Each point represents an individual caterpillar observation. The dark lines depict the mean posterior estimates, while the lighter lines illustrate 100 random draws from the posterior distribution, capturing uncertainty in the model predictions. Blue line: Plasmatocytes; Pink line: Granulocytes; Green line: Oenocytoids; Grey: Prohemocytes.

**Figure S2**: Impact of larval handling on survival following *Bacillus thuringiensis* (Bt) infection. Survival did not differ significantly between the two control groups, PBS-injected and unhandled (no-xanthotoxin) larvae (Binary logistic regression: χ² = 0.561, df = 1, p = 0.454, n = 40 per treatment), indicating that handling and injection procedures alone did not influence survival outcomes.
